# Exercise intensity as a modulator of gut microbiota and host metabolic health in obesity

**DOI:** 10.1080/19490976.2026.2661415

**Published:** 2026-04-20

**Authors:** Diana Combs, Krista Landeros, Kiara Garza, Hushyar Azari, Mostafa Abdelrahman, Kembra Albracht-Schulte

**Affiliations:** a Department of Kinesiology & Sport Management, Texas Tech University, Lubbock, TX, USA; b Center of Excellence in Obesity & Cardiometabolic Research, Texas Tech University, Lubbock, TX, USA; c Institute for One Health Innovation, Texas Tech University, Lubbock, TX, USA

**Keywords:** Exercise intensity, gut microbiota, inflammation, metabolic health, obesity, short-chain fatty acids

## Abstract

The gut microbiome is shaped by complex interactions among host, environmental, and lifestyle factors, with exercise emerging as a reported modulator. Growing evidence suggests that exercise intensity, ranging from low to high, can differentially influence gut microbial composition, diversity, and functional outputs relevant to metabolic health. This narrative review synthesizes current findings examining intensity-dependent microbial adaptations in the context of obesity. Across animal models (*n* = 17) and limited human studies (*n* = 5), moderate-intensity training (MIT) and high-intensity interval training (HIIT) produce the most consistent microbiota shifts, while low-intensity training (LIT) exerts minimal effects. Reported taxa associated with beneficial outcomes consistent across animal and human investigations include *Akkermansia* (G), and *Christensenellaceae* (F). Mechanistically, intensity-dependent alterations in microbial communities may influence obesity-related pathways through modulation of short-chain fatty acid (SCFA) and bile acid metabolism, gut barrier integrity, endotoxemia, and inflammatory signaling. HIIT and MIT are linked to improved expression of tight junction proteins (ZO-1, Claudin, Occludin), reducing circulating lipopolysaccharide (LPS), and increasing SCFA-producing taxa; thus, supporting a role for the gut microbiome in mediating exercise-induced metabolic benefits. However, inconsistent findings between species, interindividual variability, and considerable heterogeneity in exercise intervention duration across both animal (4–16 weeks) and human (3–12 weeks) studies, as well as limited longitudinal human studies, underscore the need for deeper mechanistic investigations. Future research should employ metagenomic and metatranscriptomic profiling, integrate sex- and diet-stratified longitudinal designs, and clarify causal links between exercise-responsive taxa, microbial metabolites, and host physiology. Collectively, these data highlight exercise intensity as a key determinant of gut microbiome dynamics and reinforce the need for integrative, translational approaches to define its therapeutic potential for obesity and metabolic disorders.

## Introduction

Overweight (body mass index (BMI) of 25–29.9 kg/m^2^) and obesity (BMI of ≥30 kg/m^2^), along with their complications, account for more than three million mortalities in the world annually, highlighting a crucial global burden.^1^ Individuals with central adiposity, particularly increases in visceral adiposity, have an increased risk for low-grade systemic inflammation that promotes dyslipidemia, dysglycemia, and the development of metabolic diseases such as type 2 diabetes mellitus (T2DM), metabolic dysfunction-associated steatotic liver disease (MASLD), and cardiovascular diseases (CVD).^2^ Obesity is a multifactorial disease regulated by the complex interaction between environmental, socioeconomic, genetic, and lifestyle factors.^3^ Recently, the gastrointestinal (gut) microbiome has been implicated as a key factor in obesity pathogenesis and subsequent metabolic diseases.^4^

The gut microbiome is an ecosystem made up of microorganisms including bacteria, viruses, fungi, and archaea within the gastrointestinal tract^5^ that interact with the host and play a role in regulating the immune system and metabolic functions.^6^ Although dysbiosis lacks a consensus definition, alterations in gut microbiota composition that disrupt the proportion or diversity of bacterial populations are often referred to as dysbiosis^6^^,^^7^ and have been associated with obesity and its co-morbidities.^8^^,^^9^ As a contributing interface for nutrient acquisition, the gut microbiota metabolizes dietary substrates to provide nutrient molecules to the host, dependent upon the types of substrates provided^10^ and the composition of the microbiome.^11^

Thus, the microbiome also produces metabolites that are either associated with disease development or have protective effects against disease.^12^ The gastrointestinal barrier, made up of intestinal epithelial cells interconnected by tight junctions, controls intestinal permeability and the localization of bacteria and their metabolites, such as lipopolysaccharide (LPS). Obesity is associated with increased intestinal permeability, resulting in increased circulating LPS, and systemic inflammation, which have been linked to metabolic dysfunction.^13^^,^^14^ Bacterial metabolites, specifically short-chain fatty acids (SCFAs) such as acetate, butyrate, and propionate, have a specific role in gut function and are involved in reducing inflammation, alleviating insulin resistance,^5^ decreasing intestinal permeability,^6^ and supporting metabolism.^12^

Furthermore, the gut microbiome is responsive to lifestyle interventions, including diet and exercise. Indeed, shifts in microbial composition are reported within several days of dietary improvement,^15^ independent of improved body weight.^16^ While excess energy intake contributes to overweight and obesity, dietary quality—including low fiber intake, high consumption of processed foods, and greater intake of saturated relative to unsaturated fats—has been shown to disrupt the gut microbiome and promote metabolic dysfunction. In contrast, a healthier diet, high in dietary fiber and low in processed foods, can benefit the gut microbiome and overall health.^5^^,^^17^

Exercise is recommended for the prevention and treatment of obesity and its comorbidities,^18^ given its effectiveness in reducing sedentary time, systemic inflammation, and dyslipidemia and improving insulin resistance; however, the extent of these benefits fluctuates depending on several factors.^19-21^ The FITT principle, encompassing frequency, intensity, time, and type (anaerobic vs aerobic), is a framework that suggests how exercise can vary.^22^ Intensity is a crucial factor in determining the required effort for a specific workout, but is often prescribed and reported using different physiological markers such as heart rate and VO_2_ max (Table 1). In a recent consensus statement by the American College of Sports Medicine (ACSM), 150–300 min per week of moderate intensity or 75–150 min per week of vigorous intensity is recommended to gain health benefits. Individuals with overweight and obesity may need to progress beyond 150 min per week to enhance weight loss and regulation.^23^

**Table 1.** Exercise intensity determinants.^22^^,^^24^

	Very light	Light	Moderate	Vigorous	Near max to max	HIIT
VO_2_ max	<37%	37%–45%	46%–63%	64%–90%	≥91%	70%–95%
HR max	<57%	57%–63%	64%–76%	77%–95%	≥96%	Recovery: 50%–60% Peak: 85%–95%
MET	<2.0	2.0–2.9	3–5.9	6–8.7	≥8.8	
RPE (Borg 20)	<9	9–11	12–13	14–17	≥18	
HRR	<30%	30%–39%	40%–59%	60%–89%	≥90%	

Notes: HIIT, high-intensity interval training; VO_2_, volume of oxygen consumed; HR, heart rate; MET, metabolic equivalent of task; RPE, rate of perceived exertion; HRR, heart rate reserve.

Generally, aerobic exercise aids in reducing visceral adiposity and is superior for weight loss^25^; however, there are differences in health-related outcomes between exercise intensities. High-intensity interval training (HIIT) is an enhanced form of interval training resulting in higher physical stress on the body and has gained popularity because it is time-efficient,^26^ contributes to weight loss,^27^^,^^28^ and can help those with low motivation to exercise. Moderate intensity training (MIT) and HIIT can reduce inflammation and improve glucose metabolism^29^; however, in obese mice, HIIT achieved these benefits with less time and shorter running distance.^29^ When matched for energy expenditure, HIIT is as effective as MIT at improving body composition^25^; however, evidence suggests benefits from HIIT may be more pronounced in adults with overweight or obesity than normal weight.^30^

Exercise in animal models has shown that forced and voluntary exercise can produce distinct benefits to the gut microbiome,^9^^,^^31^ evidenced by differences in the arrangement of diversity, structure, and taxonomy of the microbiome.^31^ Clusters of serum and fecal metabolites respond differentially to exercise in lean and obese individuals,^32^ and the microbiota of lean individuals demonstrates a more favorable response to exercise intervention compared to those with overweight or obesity.^33^^,^^34^ This suggests that obesity may influence metabolic responses to exercise training with the existing microbiota limiting exercise-mediated physiological improvements in an overweight population.^33^^,^^35^ Therefore, it is crucial to understand the impact of exercise on the microbiome, particularly in the context of obesity prevention and treatment, since exercise adaptations, including the microbiome, are influenced by obesity status^32^^,^^33^^,^^35^ and the intensity of the exercise.^36^ Thus, the purpose of this narrative review is to determine the effects of different exercise intensities on the gut microbiota composition in animal models and in individuals with overweight and obesity.

## Methods

This narrative review synthesizes current literature examining the impact of exercise intensity on the gut microbiome. Given the heterogeneity in study designs, populations, microbiome methodologies, and exercise prescriptions, a narrative approach was selected to allow for conceptual integration and interpretation of emerging themes. The literature search identified animal and human studies published from database inception to October 2024. Peer-reviewed, English-language articles were retrieved from the following databases: (1) MEDLINE Complete, (2) PubMed, (3) ScienceDirect, (4) Scopus, and (5) Google Scholar. Search terms included the following: Exercise (light intensity training, moderate intensity training, high intensity training, high intensity interval training, physical activity, aerobic exercise, and aerobic training), AND microbiome (gut bacteria, gut microbiota, gastrointestinal microbiome, intestinal barrier), AND obese (obesity, overweight).

Inclusion criteria comprised original research studies written in English that examined the relationship between exercise (with a focus on exercise intensity) and the gut microbiome in metabolically compromised populations. Eligible studies included animal models of obesity or metabolic dysfunction and adult human populations characterized by obesity, sedentary lifestyle, and/or increased risk of metabolic disorders. For human studies, all study designs were eligible. No restrictions were placed on study design given the emerging nature of exercise-microbiome research. Exclusion criteria were: (1) studies involving generally healthy individuals engaging in structured exercise (>3 h/week) or athletic training; (2) participants with pre-existing conditions such as cancer or autoimmune disorders or medication-treated metabolic disorders; (3) studies not investigating the gut microbiome; (4) lifestyle or multifactorial interventions; (5) association studies without exercise interventions; and (6) studies where exercise-only effects could not be isolated. Three independent reviewers determined article eligibility.

The following information was collected from each animal study: animal models, diet and composition, duration of study, food intake, ergometer, frequency, distance, speed, incline, duration of exercise, and changes in microbial community. The information collected from each human study included: number of participants, participants' age, pre-existing condition, BMI, study design, diet and diet reporting method, study duration, ergometer, intensity, frequency, speed, incline, duration of exercise, and changes in microbial community. Reported results were primarily derived from 16S rRNA gene sequencing of fecal samples, typically obtained from stool or rectal collection. A minority of studies analyzed colon or cecal tissue samples, which are specified in the table captions. Data was only included in the tables if it was reported to be significant in figures, tables, or text, or if the authors described a directional change in the bacteria in the text. Bacteria were named and reported according to the nomenclature rules established by the International Committee on Systematics of Prokaryotes.^37^

## Results

Although numerous studies have explored the impact of exercise on the gut microbiome, they have varied widely in terms of exercise type, ranging from leisure activities^38^^,^^39^ to structured exercise of varying intensities (low to high), as well as different formats (continuous or interval training), and intervention duration. Given that the objective of this review was to examine intensity-dependent changes in the gut microbiome in relation to obesity, we excluded studies focused solely on leisure activity. We stratified bacterial shifts (increases and decreases) by exercise intensity and by subject model (animal versus human). This approach resulted in a limited number of studies within each category (17 in animals; 5 in humans) that provided between group and repeated measures comparisons.

### Animal studies

The animal studies included both mouse and rat models of obesity, induced either through diet typically containing between 30% to 60% fat^40-53^ or genetic manipulation.^29^^,^^54^^,^^55^ Dietary intake was inconsistently reported, with many studies failing to quantify or report food or caloric consumption during training interventions. Among those that reported intake, most observed no significant differences between exercise and sedentary groups^29^^,^^42^^,^^47^^,^^48^^,^^50-52^; however, one HIIT protocol was associated with increased food intake under HFD conditions.^56^ Most studies employed 16S rRNA sequencing of fecal samples, although one used a targeted approach^53^ and a few analyzed colon and/or cecum tissue^49^^,^^50^^,^^53^^,^^54^ directly. Exercise interventions in these models consistently influenced microbial populations within several phyla, notably Bacillota, Pseudomonadota, Actinomycetota, Bacteroidota, Mycoplasmatota, Verrucomicrobiota, and Euryarchaeota;^40-53^^,^^55^^,^^57^ however, most significant microbial shifts were observed at the family and genus levels. Among the studies conducted in animals, bacteria within the Bacillota phylum were the most affected in all exercise intensity interventions, with HIIT resulting in the greatest magnitude of change in bacteria (increased or decreased).

Among the three studies conducted at low intensity (Table 2), microbial shifts were observed in the Bacillota, Pseudomonadota, Actinomycetota, Bacteroidota, and Euryarchaeota phyla.^40^^,^^41^^,^^54^ However, changes in the bacterial microbiota were inconsistent across studies. For example, *Lactobacillus* (G), *Streptococcus* (OTU115) (G), and *Streptococcaceae* (F)—all members of the class Bacilli within the Bacillota phylum—were significantly decreased following exercise, while bacteria within *Clostridia* (C), also within the Bacillota phylum, were significantly increased.^40^^,^^41^

**Table 2.** Impact of light-intensity training on gut microbiota of male rodents in between-group comparisons.

				Exercise intervention				Impact on microbial community		
Animal model	Diet	Control	Duration (weeks)	Ergometer	Frequency (days/wk)	Speed/intensity	Session time (min)	Increased	Decreased	Ref.
db/db (*n* = 10)	Chow	db/db + SED (*n* = 9)	6	Treadmill	5	2.87 m/min (189 m/session)	66	Bacillota (*P*) *↑*Clostridium leptum (C-IV)*^a^ *↑*Clostridium cluster (C-I)*^a^	Pseudomonadota (P) *↓*Enterobacteriaceae* (F)^a^ Actinomycetota (P)^a^ *↓*Bifidobacterium spp* (G)^a^ Bacteroidota (P) *↓*Bacteroides/Prevotella spp.* (G)^a^ Euryarchaeota (P) *↓*Methanobrevibacter spp.*(G)^a^ Bacillota (P) *↓*Clostridium cluster (C-XI)*^a^	54
C57BL/6 J (*n* = 10)	HFD (60%)	HFD + SED (*n* = 10)	16	Running wheel	5	7 m/min	60	*↑Bacillota (P) *↑*Peptostreptococcaceae* (F) Pseudomonadota (P) *↑*Phyllobacteriaceae* (F) *↑*Pseudomonadaceae* (F) *↑*Caulobacteraceae* (F) *↑*Burkholderiaceae* (F) Bacteroidota (P) *↑*Flavobacteriaceae* (F) *↑*Sphingobacteriaceae* (F)	Bacillota (P) *↓*Streptococcaceae* (F) *↓*Streptococcus (OTU115)* (G) *↓Bacteroid*ota* (P) *↓*Porphyromonadaceae* (F)	40
Wild type	HFD 60%	HFD + SED	15	Treadmill	5	7 m/min	60	Bacillota (*P*) *↑*Lachnospiraceae_Nk4A136_group* (G)	Bacillota (P) *↓*Lactobacillus* (G) *↓*Faecalibaculum* (G) Actinomycetota (P) *↓*Coriobacteriaceae_UCG-002* (G)	41

Notes: P, phylum; C, class; F, family; G, genus. Arrows indicate reported changes: ↑, increased; ↓, decreased. Arrows without *indicate non-significant changes described in the text. Taxa (phyla) without arrows are listed for clarification. Bacterial names follow the most recent International Code of Nomenclature of Prokaryotes. HFD, high-fat diet; SED, sedentary; N.C., no change; SD, normal diet; db/db, type 2 diabetic; db/+, control *significant change, *p* < 0.05. ^a^from cecum; *Clostridium* clusters (I, IV, XI) are legacy groupings from the original studies; according to current taxonomy, most members are now reclassified within the families *Ruminococcaceae* and *Lachnospiraceae*.

According to the eight studies conducted at moderate intensity (Table 3), bacterial changes were observed within the Bacillota, Pseudomonadota, Actinomycetota, Bacteroidota, Mycoplasmatota, and Verrucomicrobiota phyla.^29^^,^^42-46^^,^^53^^,^^55^ However, shifts in the gut microbiota were again inconsistent across studies. Interestingly, while several genera and species within the Bacillota phylum tended to increase,^42-46^^,^^55^ the phylum showed a decreased abundance in one study.^45^ Conversely, the Bacteroidota phylum increased in some studies,^43^^,^^45^while specific taxa such as the species *Prevotella copri* (S)^53^ and *Parabacteroides* (G)^43^ were decreased. Within the Mycoplasmatota phylum, *Anaeroplasma* (G) increased, while other members of the phylum decreased.^43^ The Verrucomicorbiota phylum and the *Akkermansia* (G) within it both increased.^44^ The one study investigating high intensity training (Table 4), reported bacterial changes in the Bacillota, Actinomycetota, Psudomondaota, Campylobacterota, and Bacteroidota phyla. Consistent with changes seen in LIT and MIT studies, the *Lactobacillus* (G) (Bacillota phylum) and *Parabacteroides* (G) (Bacteroidota phylum) were decreased.^47^ Among the seven studies employing HIIT (Table 5), exercise consistently modulated gut microbiota phyla including Bacillota, Cyanobacteriota, Thermodesulfobacteriota, Bacteroidota, Mycoplasmatota, and Verrucomicrobiota.^29,48–53^ While other intensity levels were associated with inconsistent changes at the genus and species levels, some consistent trends emerged with HIIT: *Prevottella* (G) of the *Bacteroidota* phylum^48^^,^^51^ and *Anaeroplasma* (G) of the Mycoplasmatota phylum^48^^,^^50^ each increased in two separate studies.

**Table 3.** Impact of moderate intensity training on gut microbiota of male rodents in between-group comparisons.

				Intervention				Impact on microbial community		
Animal model	Diet	Control	Duration (weeks)	Ergometer	Frequency (days/wk)	Speed/intensity	Time (min)	Increased	Decreased	Ref.
Zucker rats^a,e^ (*n* = 3)	N/A	Wistar rats^f^ (*n* = 3)	4	Treadmill	5	Corresponded to MLSS:12.5 m/min (Zucker) 20 m/min (Wistar)	30	Bacillota (P) *↑*Ruminococcus gnavus* (S) *↑*Streptococcus alactolyticus* (S) Pseudomonadota (P) *↑*Aggregatibacter* *Pneumotropica* (S) Actinomycetota (P) *↑*Bifidobacterium animalis* (S) *↑*Bifidobacterium* *Pseudolongum* (S)	N.C.	55
C57BL/6 mice (*n* = 10)	HFD (60%)	HFD + SED (*n* = 10)	8	Treadmill	5	50% *V*_max_	30	Bacillota (P) *↑*Vagococcus* (G)	Pseudomonadota (P) *↓*Proteus*(G)	42
Sprague-Dawley rats (*n* = 8)	HFD (60%)	HFD + SED (*n* = 10)	8	Treadmill	6	20 m/min (0% grade)	60	Bacillota (P) *↑*Erysipelotrichaceae_-UCG_003* (G) *↑*Ruminiclostridium*_5 (G) *↑*Eisenbergiella* (G) Mycoplasmatota (P) *↑*Anaeroplasma* (G) Actinomycetota (P) *↑*Micrococcaceae* (F)	Bacillota (P) ↓*Romboutsia* (G) *↓*Rumminiclostridium_1* (G) *↓*Clostridium_sensu_stricto_1* (G) *↓*Peptostreptococcaceae* (F) *↓*Clostridiaceae_1* (F) Bacteroidota (P) *↓*Parabacteroides* (G)	43
C57BL/6J Mice (*n* = 6)	HFD (60%)	HFD + SED (*n* = 6)	12	Treadmill	5	12 m/min (at 76% VO_2_ max)	45	Bacillota (P) *↑*Ruminococcaceae_UCG-014* (G) *↑Verrucomicrobia (P) *↑*Akkermansia* (G)	N.C.	44
BL6 mice (*n* = 10)	HFD (60%)	HFD SED (*n* = 10)	8	Treadmill	6	10 13 m/min^Ŧ^ (0% grade)	40–50^Ŧ^	*↑Bacteroidota (P) Bacillota (P) *↑ *L. acidophilus* (S)	*↓Bacillota (P)	45
Zucker Rats^a^ (*n* = 12)	SD	SED (*n* = 12)	10	Treadmill	5	12 m/min (at 0% grade)	51	N.C.	N.C.	29
Wistar rats^d^ (*n* = 8)	30% animal fat, 25% fructose, 45% basic animal food	Diabetic + SED (*n* = 8)	10	Treadmill	N/A	50%–60% VO_2_ max	30	N.C.	Bacteroidota (P) *↓*Prevotella copri* (S)^c^	53b
C57BL/6 mice (NAFLD induced)	HFD	HFD + SED	6	Treadmill	5	Acclimation: 10 cm/s Acceleration: +1cm/s until 20 cm/s Maintenance: 20 cm/s Deceleration: −4 cm/s until 0 cm/s	Acclimation: 5 min Acceleration: 10 min Maintenance: 20 min Deceleration: 5 min	Bacillota (P) *↑*Dubosiella* (G)	N.C.	46

Notes: P, phylum; O, order; F, family; G, genus; S, species. Arrows indicate reported changes: ↑, increased; ↓, decreased. Arrows without *indicate non-significant changes described in the text. Taxa (phyla) without arrows are listed for clarification. Bacterial names follow the most recent International Code of Nomenclature of Prokaryotes. HFD, high-fat diet; SED, sedentary; MLSS, maximal lactate steady state; N.C., no change; SD, standard diet; *V*_max_, maximum velocity. ^a^Genetically obese mice model; ^b^primers selected for Prevotella copri, Akkermansia muciniphila, and Butyrivibrio fibrisolvens; ^c^from cecum; ^d^diet-induced diabetes; ^e^Bacteroides acidifaciens (S) was significantly more abundant in Zucker rats than in Wistar rats at baseline; ^f^Ruminococcus flavefaciens (S) had significantly greater abundance in Wistar rats than Zucker rats at baseline; ^Ŧ^progressively increased intensity over study duration; *significant change, *p < *0.05.

**Table 4.** Impact of high-intensity training on gut microbiota of male rodents in between-group comparisons.

				Intervention				Impact on microbial community		
Animal model	Diet	Control	Duration (weeks)	Ergometer	Frequency (days/wk)	Speed/intensity	Time (min)	Increased	Decreased	Ref.
C57BL/6JNarl mice (*n* = 7)	HFD (60%)	HFD + SED (*n* = 6)	16	Treadmill	5	18 m/min (at 0% grade)	30	Bacteroidota (*P*) *↑*Odoribacter* (G) *↑*AF12* (G) Pseudomonadota (*P*) *↑*Aggregatibacter* (G) Campylobacterota (*P*) *↑*Helicobacter* (G) Bacillota (*P*) *↑*Turicibacter* (G)	Bacteroidota (*P*) *↓*Parabacteroides* (G) Actinomycetota (*P*) *↓*Bifidobacterium* (G) Campylobacterota (*P*) *↓*Flexispira* (G) Bacillota (*P*) *↓*Lactobacillus* (G) *↓*Lactococcus* (G)	47

Notes: P, phylum; G, genus. Arrows indicate reported changes: ↑, increased; ↓, decreased. Arrows without

^*^ indicate non-significant changes described in the text. Taxa (phyla) without arrows are listed for clarification. Bacterial names follow the most recent International Code of Nomenclature of Prokaryotes. HFD, High-fat diet; SED, sedentary; *significant change, *p < *0.05.

**Table 5.** Impact of high intensity interval training on gut microbiota of male rodents in between-group comparisons.

				Intervention			Impact on microbial community		
Animal model	Diet	Control	Duration (weeks)	Ergometer	Frequency (days/wk)	Protocol	Increased	Decreased	Ref.
Wistar rats (*n* = 8)	HFD 43%	HFD + SED (*n* = 12)	12	Treadmill	4	6 × (3 min at 10 m/min and 4 min × 18 m/min)	Bacteroidota (*P*) *↑*Prevotella* (G) Mycoplasmatota (*P*) *↑*Anaeroplasma* (G) Cyanobacteriota (*P*) *↑*Cyanobacteria YS2* (G)	Bacillota *↓*Clostridiales* spp. (G)	48
Zucker Rats^a^ (*n* = 12)	SD	SED (*n* = 12)	10	Treadmill	5	6 × (4 min at 18 m/min and 3 min 10 m/min) 0% grade	N.C.	N.C.	29
C57 BL/6 mice (*n* = 8)	HFD (45%)	HFD + SED (*n* = 8)	6	Treadmill	3	6 × (2 min at 17–22 m/min^Ŧ^ + 2 min rest) 5% grade	Bacteroidota (*P*) *↑Bacteroidales (O)^b,c^ Bacillota (*P*) *↑ Bacilli (C)^b^ *↑ *Dorea* (G)^b,c^	Bacillota (*P*) *↓*Lachnospiraceae* (F)^b^ *↓*F.Clostridium* (S)^b^ *↓*Clostridiaceae* (F)^c^	49
Wistar rats (*n* = 10)	HFD (45%)	HFD + SED (*n* = 12)	12	Treadmill	4	6 × (3 min at 10 m/min and 4 min at 18 m/min)	*↑ Mycoplasmatota (*P*)^b^ ↑ *Anaeroplasmataceae* (F)^b^ *↑ *Anaeroplasma* (G)^b^ Bacillota (*P*) *↑*Christensenellaceae* (F)^b^	N.C.	50
Wistar rats (*n* = 8)	HFD (60%)	HFD + SED (*n* = 8)	10	Treadmill	5	5–12^Ŧ^ × (30 s at 29–36 m/min^Ŧ^ with 60 s at 13 m/min) up to 90% VO_2_ max, at 0% grade	*↑Bacteroidota (*P*) *↑*Bacteroides* (G) *↑*Prevotella* (G) Bacillota (P) *↑*Roseburia* (G)	*↓Bacillota (P) *↓*Lactobacillus* (G) Bacteroidetes (P) *↓*Alistipes* (G) Actinomycetota (P) *↓*Bifidobacterium*(G) Thermodesulfobacteriota (P) *↓*Bilophila* (G)	51
C57BL6 (*n* = 15)	Western diet (41.91%)	Western diet + SED (*n* = 15)	6	Treadmill	3	5 × (60 s at 90% *V*_max_ and 60 s at 60% *V*_max_)	Bacillota (P) *↑*Eubacterium xylanophilum group* (G) *↑*UBA1819* (G)	N.C.	52
Wistar rats^d^ (*n* = 8)	30% animal fat, 25% fructose, 45% basic animal food	SED (*n* = 8)	10	Treadmill	5	4 × (60 s at 30%–35% VO_2_ and 3 min at 90% VO_2_)	Verrucomicrobia (P) *↑*Akkermansia muciniphila* (S)^c^ Bacillota (P) *↑*Butyrivibrio fibrisolvens* (S)^c^	Bacteroidota (P) *↓*Prevotella copri* (S)^c^	53e

Notes: P, phylum; C, class; F, family; G, genus; S, species. Arrows indicate reported changes: ↑, increased; ↓, decreased. Arrows without *indicate non-significant changes described in the text. Taxa (phyla) without arrows are listed for clarification. Bacterial names follow the most recent International Code of Nomenclature of Prokaryotes. HFD, High-fat diet; SED, sedentary; N.C., no change; SD, standard diet; *V*_max_, velocity max. ^a^Genetically obese mice model; ^b^from colon; ^c^from cecum; ^d^diet-induced diabetes; ^e^primers selected for *Prevotella copri*, *Akkermansia muciniphila*, and *Butyrivibrio fibrisolvens*; ^Ŧ^progressively increased intensity over study duration; *significant change, *p*  <  0.05.

Three studies employed a repeated measures design in their investigation (Table 6).^49^^,^^51^^,^^55^ In Zucker rats undergoing MIT, increases were observed in *Pseudomonas* (G) within the Pseudomonadota phylum and *Lactobacillus* (G) within Bacillota,^55^ which were unique compared to changes seen in between group comparisons. In HIIT studies, increases in Bacteroidales (O) from the Bacteroidota phylum were reported,^49^ which was consistent with between group comparisons.^49^ In the other study, increases in *Roseburia* (G) from the Bacillota phylum were accompanied by decreases in multiple genera within Bacteroidota, including *Prevotella*, *Alistipes*, and *Bacteroides*, as well as reductions in *Lactobacillus* and *Ruminococcus* within Bacillota.^51^

**Table 6.** Impact of training on gut microbiota of male rodents in repeated measures comparisons.

			Intervention					Impact on microbial community		
Animal model	Diet	Duration (weeks)	Type	Ergometer	Frequency (days/wk)	Intensity	Time	Increased	Decreased	Ref.
Zucker rats^a^ (*n* = 3)	N/A	4	MIT	Treadmill	5	12.5 m/min^−1^	30 min	Pseudomonadota (P) *↑*Pseudomonas* (G) Bacillota (*P*) *↑*Lactobacillus* (G)	N.C.	55
C57BL/6 mice (*n* = 8)	HFD (45%)	6	HIIT	Treadmill	5	17–22 m/min^Ŧ^ (at 5% grade)	60 min (2 min run + 2 min rest)	Bacteroidota (P) *↑Bacteroidales (O)	N.C.	49
Wistar Rats (*n* = 8)	HFD (60%)	10	HIIT	Treadmill	5	13–36 m/min^Ŧ^ (up to 90% VO_2,_ at 0% grade)	5 × 30 s (29–36 m/min^Ŧ^) with 60 s active recovery	Bacillota (P) *↑*Roseburia* (G)	Bacteroidota (P) *↓*Prevotella* (G) *↓*Alistipes* (G) *↓*Bacteroides* (G) Bacillota (P) *↓*Lactobacillus* (G) *↓*Ruminococcus* (G)	51

Notes: P, phylum; O, order; G, genus. Arrows indicate reported changes: ↑, increased; ↓, decreased. Taxa (phyla) without arrows are listed for clarification. Bacterial names follow the most recent international Code of Nomenclature of Prokaryotes. HFD, high-fat diet; MIT, moderate intensity training; HIIT, high intensity interval training; N.C., no change. ^a^Genetically obese mice model; ^Ŧ^progressively increased intensity over study duration; *significant change, *p* < 0.05.

### Human studies

Only five human studies met inclusion criteria for this review. None employed low-intensity exercise interventions. All interventions instructed participants to maintain their habitual diet. Although diet was typically recorded, assessment methods varied and were often limited to 3–7-d food diaries collected pre- and/or post-intervention. Some studies relied solely on baseline dietary assessments and assumed dietary stability throughout the intervention.^58^^,^^59^ Only one study accounted for the intake of energy-yielding or gut–microbiota affecting nutrients in their analysis.^60^ All studies employed 16S rRNA sequencing of fecal samples,^27^^,^^59-61^ although one used a targeted approach.^58^ In the study using moderate intensity intervention (Table 7), increases were observed in the Bacillota, Actinomycetota, and Verrucomicrobiota phyla, while decreases occurred in the Pseudomonadota, Bacteroidota, and Thermodesulfobacteriota phyla.^60^ MIT increased the Verrucomicrobiota phylum and *Akkermansia* (G) within.^60^ Among the two high intensity training studies (Table 8), there were increases in the Bacillota and Actinomycetota phyla.^58^^,^^61^ Interestingly, the *Bifidobacterium* (G) (Actinomycetota phylym) reported an increase in humans,^58^ but was reported to decrease in animal models.^47^ Finally, among the two HIIT studies (Table 9), there was an increase in Actinomycetota phylum,^27^ but bacterial changes in the Bacillota phylum were inconsistent.^27^^,^^59^

**Table 7.** Impact of moderate intensity training on gut microbiota of humans in repeated measures comparisons.

						Intervention					Impact on microbial community		
*N* (M/F)	Age (years)	Pre-existing condition	BMI (kg/m^2^)	Duration (weeks)	Diet/ record	Type	Ergometer	Frequency (days/wk)	Intensity	Time (min)	Increased	Decreased	Ref.
17 (F)	36.8 ± 3.9	SED + overweight/obesity	31.8 ± 4.4	6	Habitual *ad libitum* diet/food diaries for 3 d pre/post-intervention	LIT: wk 1–2 LIT or MIIT (alternate every other session): wk 3–4 MIIT: wk 5–6	Cycle	3	60 rpm	40–60^Ŧ^	*↑Verrucomicrobiota(*P*) *↑*Verrucomicrobiaceae* (F) *↑*Akkermansia* (G) Bacillota(*P*) *↑*Dorea* (G) *↑*Anaerofilum* (G) Actinmycetota(*P*) *↑*Bifidobacteriaceae* (F)	*↓Pseudomonadota(*P*) *↓unidentified *Porphyromonadaceae* (G) *↓unidentified *Enterobacteriaceae* (G) Bacteroidota(*P*) *↓*Odoribacter* (G) Thermodesulfobacteriota (*P*) *↓unidentified *Desulfovibrionaceae*(G)	60

Notes: P, phylum; F, family; G, genus. Arrows indicate reported changes: ↑, increased; ↓, decreased. Taxa (phyla) without arrows are listed for clarification. Bacterial names follow the most recent International Code of Nomenclature of Prokaryotes. SED, Sedentary; LIT, light intensity training; MIIT, moderate intensity interval training; rpm, rotations per minute. ^Ŧ^Progressively increased intensity over study duration; *significant change, *p* < 0.05.

**Table 8.** Impact of high-intensity training on gut microbiota of humans in repeated measures comparisons.

						Intervention					Impact on microbial community		
*N* (M/F)	Age (years)	Pre-existing conditions	BMI (kg/m^2^)	Duration (weeks)	Diet/ record	Type	Ergometer	Frequency (days/wk)	Intensity	Time (min)	Increased	Decreased	Ref.
14 (7/7)	51 ± 11	SED + obese	34.9 ± 4.9	8	Habitual *ad libitum* diet/7-d food diary pre/post-intervention	HIT	Cycle	2–4 ^Ŧ^	65%–85%^Ŧ^ HRR	50	Bacillota (P) *↑*Anaerostipes* (G) *↑*Lachnospiraceae FCS020 group* (G) *↑*Ruminococcus gauvreauii group* (G)	N.C.	61
9 (F)	20.74–27	Low activity + overweight	27.6 ± 1.6	10	Habitual *ad libitum* diet/72-h recall pre-intervention	M-HIT^Ŧ^	Treadmill	3	55%–75%^Ŧ^ HRR	30–45 ^Ŧ^	Actinomycetota (P) *↑*Bifidobacterium* (G)	N.C.	58

Notes: P, phylum; F, family; G, genus; S, species. Arrows indicate reported changes: ↑, increased; ↓, decreased. Taxa (phyla) without arrows are listed for clarification. Bacterial names follow the most recent International Code of Nomenclature of Prokaryotes. MIT, moderate intensity training; HIT, high-intensity training; SED, sedentary. HRR, heart rate reserve; VO_2_, volume of oxygen consumed; N.C., no change. ^Ŧ^Progressively increased intensity over study duration; *significant change, *p*  <  0.05. ^a^Primers used targeted *Lactobacillus* and *Bifdobacterium* only.

**Table 9.** Impact of high intensity interval training on gut microbiota of humans in repeated measures comparisons.

						Intervention					Impact on microbial community		
*N* (M/F)	Age (years)	Pre-existing conditions	BMI (kg/m^2^)	Duration (weeks)	Diet/record	Type	Ergometer	Frequency (days/wk)	Intensity	Time (min)	Increased	Decreased	Ref.
15 (M)	31 ± 2	Overweight	29.6 ± 2.7	3	Habitual *ad libitum* diet/ 6-month FFQ pre-intervention	HIIT	Cycle	3	Sprint: VO_2_ peak Recovery: rest	8–12^Ŧ^ × 60 s bouts interspersed by 75 s rest	N.C.	Bacillota (P) *↓Subdoligranulum (G)	59
8(M): Cycle 8(M) Treadmill	Cycle: 52.9 ± 10.3 Treadmill: 55.7 ± 9.3	Overweight/obese	Cycle: 30.7 ± 2.8 Treadmill: 29.2 ± 1.5	12	Habitual *ad libitum* diet/7-d food diary pre/post-intervention	HIIT	Cycle Or Treadmill^a^	3	Sprint:80%–85% HR max Recovery: 40%–45% HR max	Cycle: 10 × 45 s followed by 90 s of active recovery Treadmill: 9 × 45 s followed by 90 s of active recovery	Bacteroidota(*P*) *↑Rikenellaceae(F) Bacillota(*P*) *↑Clostridiaceae(F) ↑Christensenellaceae (F) Actinomycetota(*P*) *↑Actinymycetaceae (F)	N.C.	27

Notes: P, phylum; F, family; G, genus. Arrows indicate reported changes: ↑, increased; ↓, decreased. Arrows without *indicate non-significant changes described in the text. Bacterial names follow the most recent International Code of Nomenclature of Prokaryotes. Taxa (phyla) without arrows are listed for clarification. FFQ, food frequency questionnaire; HIIT, high-intensity interval training; VO_2_, volume of oxygen consumed N.C., no change. ^a^Treadmill and Cycle protocols were tested to ensure they were isoenergetic; ^Ŧ^progressively increased intensity over study duration; *significant change *p*  <  0.05.

## Discussion

**Figure 1.**
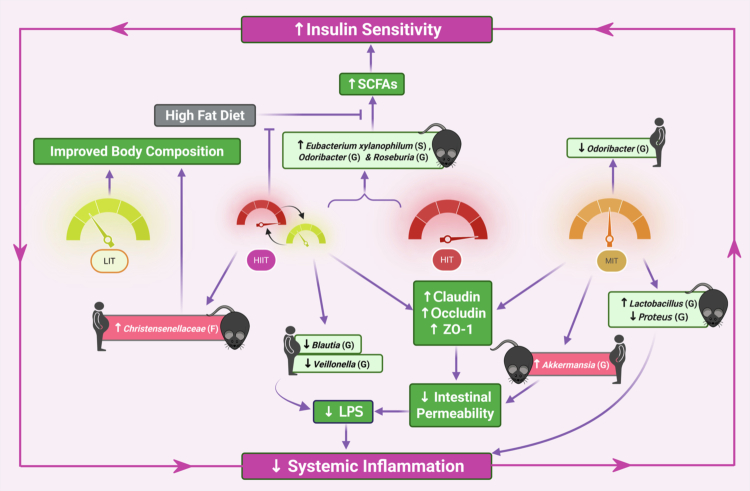
Schematic illustration of how exercise intensity can affect the gut microbiota composition and consequent metabolic outcomes in the context of obesity. Low (LIT), moderate (MIT), high-intensity (HIT), and high-intensity interval training (HIIT) influence distinct bacterial taxa causing higher expression of tight-junction proteins, including claudin, occludin, and ZO-1, reduced intestinal permeability, and reduction in circulating lipopolysaccharides (LPS). Increased beneficial microbial composition is linked with increased short-chain fatty acid (SCFA) production, promotion of insulin signaling, and decreased systemic inflammation, with few taxa associated with improved body composition. Created in BioRender.com.

The gut microbiome is shaped by a complex interplay of host-related factors (age, sex, genetics, and perinatal exposures), environmental influences, disease states, stress, and lifestyle factors—particularly diet and exercise.^62^ While exercise is increasingly recognized as a modulator of microbial composition, diversity, and functional outputs, its effects remain highly debated. Variability in exercise type (aerobic, muscular fitness, flexibility, neuromotor), duration, intensity (light, moderate, high), and frequency,^22^ may elicit distinct physiological and psychological stress responses, producing heterogeneous microbial outcomes.^31^^,^^63^ Given the rising prevalence of obesity and the blunted exercise response observed in this population,^64^ understanding intensity-dependent effects is of relevance for guidance/prescription in this population. Here, we report that exercise intensity influences microbiome shifts in the context of obesity, with HIIT producing the most consistent influence, MIT showing moderate alterations, and LIT causing minimal changes, though these findings are mostly from animal studies, and translation to humans remains limited and inconsistent. Mechanistically, shifts in gut microbial composition in response to varying exercise intensities may influence multiple pathways implicated in obesity and related metabolic disorders (Figure 1).

### Taxonomic responses to exercise intensity

**Figure 2.**
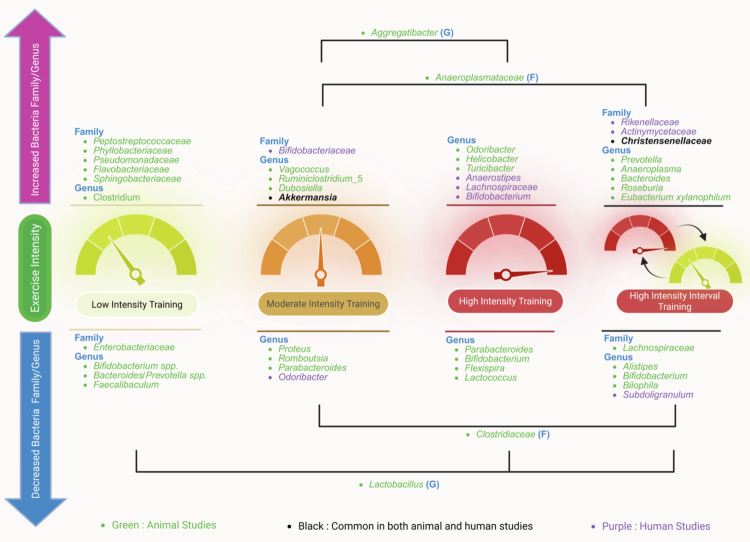
Summary of bacterial families and genera that are altered by different exercise intensities across animal and human studies. Low, moderate, and high-intensity training, as well as high-intensity interval training, demonstrate specific microbial profiles, with beneficial taxa such as Akkermansia enriched mostly at higher intensities. Green: animal studies; purple: human studies; black: common in both animal and human. Created in BioRender.com.

Physical activity induces physiological stress on the body that changes gut microbial composition^65^; though few bacterial taxa respond consistently to exercise (Figure 2). Generally, the *Akkermansia* genus increased in relative abundance in response to MIT in both mice and humans,^44^^,^^60^ while the specific species, *Akkermansia munciniphila,* a mucin-degrading bacteria linked to improved barrier integrity and metabolic health,^66^^,^^67^ increased in response to HIIT in rats.^53^ Human studies yield controversial results, with a recent systematic review finding four studies showed an increased abundance of *Akkermansia muciniphila* following low to moderate aerobic training, but three others showed no change or a decreased abundance.^68^

It has been well established that *Bifidobacterium* exert positive effects on health.^69^ Though, the *Bifidobacterium* genus response appears host- and context-dependent. In animals there was a reported decrease in abundance,^47^ whereas in humans an increased abundance was observed^58^ following HIT. Furthermore, *Bifidobacterium* spp. increased in non-diabetic mice but remained low in diabetic mice after LIT, indicating that lighter intensity exercise was insufficient in inducing beneficial shifts in *Bifidobacterium* spp. in metabolically challenged conditions.^54^ However, the absence of change in this model does not constitute evidence that LIT is ineffective more broadly. Collectively, opposing effects of HIT on *Bifidobacterium* (G) in animal and human studies may be because of differences in baseline microbiota composition, host metabolic status, study duration, and the physiological stress used in the forced HIT method.^54^

Similar context-dependent patterns are evident in other taxa*. Prevotella* (G) from the Bacteroidota phylum and *Anaeroplasma* (G) from the Mycoplasmatota phylum increased in response to HIIT in animal models in two independent studies. *Prevotella* is positively associated with physical activity and high fiber diets and has been negatively correlated with weight and fat mass gain.^48^ However, its role in metabolic health is strain dependent. For instance, *Prevotella copri* has been implicated in insulin resistance in obese rodent models and humans, underscoring the need for cautious interpretation of *Prevotella* enrichment as inherently beneficial.^70^^,^^71^ Further, there is little information on the association of *Anaeroplasma* abundance with physical activity, studies suggest it may have potential as an anti-inflammatory probiotic treatment for obesity and related intestinal issues.^48^^,^^51^

In addition to these genera, *Christensenellaceae* (F) was increased by HIIT in both animal and human models^27^^,^^50^ and has been consistently shown to be significantly enriched in individuals with a healthy BMI.^72^ Due to its strong associations with health, *Christensenellaceae* has been proposed for use as a therapeutic probiotic.^72^ Together, these taxa-level findings highlight that exercise intensity may influence specific microbial groups; however, these responses are strongly modulated by host context and study design.

Interpretation of taxa-level findings must also consider methodological complexity. Discrepancies between repeated measures and within group analyses results of the three studies that employed these methods revealed differences in the reported changes in relative abundance and highlighted important methodological and biological complexities. Bacteria that showed consistent trends (*Roseburia*, *Alistipes*, *Lactobacillus*) across both types of analyses demonstrate a strengthened likeliness of their association with the exercise intervention; however, the bacteria that presented with opposite changes (*Prevotella* and *Bacteroides*) or changes unique to repeated measures analysis indicate there may be variability due to individual differences in mice models, environmental factors, or methodological discrepancies.^51^ These inconsistencies depict the limitations of single-timepoint or simplified analyses, which may obscure true exercise-induced microbial shifts.^73^ Moreover, variability in sequencing targets, sample processing, and bioinformatic pipelines likely contributes to inconsistent findings across studies,^74^ highlighting the need for consensus frameworks and standardized reporting guidelines in exercise-microbiome research.

Beyond analytic variability, conceptual limitations must also be acknowledged. Reporting exercise-induced shifts in microbial taxa solely through relative taxonomic abundance may not accurately capture the functional impact of exercise. Taxonomic shifts do not necessarily translate to functional alterations, as substantial functional redundancy exists within microbial communities—phylogenetically distinct taxa harbor similar genes and perform overlapping metabolic functions.^75^ Indeed, metagenomic studies demonstrate that although microbial taxonomic composition varies among individuals, overall gene content and functional pathways remain relatively conserved.^76^ Thus, interpreting exercise-related adaptations based only on taxonomic changes may overlook meaningful functional stability or shifts that would be better detected through metagenomic or metatranscriptomic analyses. Notably, within this review, no animal study and only one human study incorporated such functional approaches.^60^ Furthermore, integrating metagenomics with metabolomics would provide a more comprehensive framework for identifying exercise-induced microbial and metabolic adaptations,^77^ yet only a limited number of animal studies reported metabolomics outcomes, namely the impact on SCFAs.^29^^,^^48^^,^^53^

Accordingly, future investigations should prioritize functional profiling through shotgun metagenomics and metatranscriptomics rather than relying solely on amplicon-based community composition. The integration of metagenomics, metatranscriptomics, and metabolomics is ideal to resolve the functional consequences of exercise-induced shifts in the gut ecosystem.

### Microbial metabolites, barrier function, and dietary context

Functional interpretation is particularly important when considering microbial metabolites. Short-chain fatty acids (SCFAs)—mainly butyrate, acetate, and propionate—are key microbial metabolites linking exercise to gut and metabolic health.^78^ For instance, both intestinal microbiota richness and increased butyrate, a SCFA that promotes gut epithelium integrity and local energy metabolism,^79^ are significantly associated with improved cardiorespiratory fitness (V̇O2peak) in healthy individuals.^80^

Exercise intensity and duration appear to differentially influence SCFA-producing taxa. Animal studies show that HIT (consistent or interval), increases SCFA producers such as Eubacterium xylanophilum,^52^ *Odoribacter,*^47^ Roseburia^51^ and AF12,^47^ and improves insulin sensitivity^53^ while MIT increases taxa like Lactobacillus^55^ that support mucosal immunity and more effectively reduce inflammatory markers.^81^ However, translation to humans remains inconsistent, as decreased *Odoribacter*^60^ after MIT and reduced Subdoligranulum after short-term HIIT^59^ have been reported. Intervention duration further modifies outcomes, with benefits more evident following ≥6–10-week protocols.

The magnitude of these microbial and metabolic responses is strongly influenced by dietary context. A HFD can blunt SCFA production and limit exercise-induced benefits in mice.^33^^,^^34^ LIT altered intestinal microbiota but failed to mitigate the negative effects of 16 weeks of HFD (60% calories from fat) feeding in C57BL/6 mice.^40^ Similarly, MIT,^29^^,^^42^ HIT,^47^ and HIIT^29^ did not fully counteract HFD-induced alterations in animal models. Although HIIT partially restored distal gut and fecal microbiota disrupted by obesity and/or HFD, the magnitude of these changes was modest compared with those driven by diet.^49^ This suggests that while diet exerts a primary, direct influence on gut microbiota composition, exercise—despite its broad physiological benefits—plays a secondary role.

Microbial shifts may also influence host metabolism through bile acid receptor signaling pathways, linking microbiota composition to glucose metabolism, gut barrier function, and lipid metabolism.^82^ For example, *Bacteroides*, which decreased in response to LIT^54^ but increased in response to HIIT^51^ in rodent models, is involved in bile acid metabolism.^83^ Additionally, the gut-brain axis, a bidirectional pathway between the gut and the central nervous system, may be influenced by bacterial metabolites and affect neural signaling, mood, and behavior.^84^ This warrants further research in the context of exercise and obesity.

Given the dominant role of diet on the gut microbial composition, inconsistencies in dietary assessment represent a critical methodological limit. Future investigations should prioritize rigorous approaches to disentangle the independent and interactive effects of diet and exercise on microbial and metabolic outcomes. Controlled feeding studies, short-term dietary standardization prior to stool collection^85^ (i.e., a 3-d standardized diet that is repeated prior to each collection time point), and statistical adjustment for total energy, fiber, and macronutrient intake would strengthen causal inference in humans. Additionally, crossover or factorial designs (diet x exercise) may help clarify whether observed microbial shifts are directly attributable to exercise or mediated through diet-induced changes in substrate availability.^86^

### Body composition and metabolic outcomes

Exercise-induced improvements in body composition, particularly increases in lean mass, have been linked to gut microbiota shifts.^64^ Correlation studies report that *Ruminococcaceae*, *Prevotella*, and *Lachnospira* may play a part in modulating body composition and cardiovascular health.^43^ In rodent models, LIT,^40^^,^^41^ MIT,^29^^,^^42^^,^^43^ and HIIT^29^ all improved body composition relative to HFD sedentary controls, with HIIT producing greater reductions in total fat mass and visceral adipose tissue than MIT.^29^ However, the impact of exercise on body composition and other health outcomes and correlation to changes in microbiota composition may be dependent on intervention duration and is complex.^49^^,^^50^ For example, six weeks of LIT-MIIT in females with overweight/obesity altered gut microbiome composition and functional capacity without marked changes in systemic metabolites or body composition.^60^ Conversely, increases in *Lactobacillus* were inversely correlated with changes in body weight and BMI and positively associated with improvements in aerobic capacity (VO_2_peak), while increases in *Bifidobacterium* were correlated with greater weight loss and reduced BMI following 10 weeks of M-HIT training in overweight females.^60^ Moreover, short-term HIIT (3 weeks) did not alter fat mass or microbiota composition in overweight males despite improvements in cardiorespiratory fitness;^59^ however, the brief duration may have limited the ability to detect microbiome adaptations.

### Inflammation and barrier integrity

Microbial alterations may also mediate exercise-related reductions in inflammation. Exercise may reduce systemic endotoxemia by increasing Bacteroidota and reducing LPS-producing taxa like *Proteus* (G), which lowers circulating LPS and its complexation with lipopolysaccharide-binding protein (LBP), ultimately attenuating TLR4-mediated inflammation.^87^ LPS binds directly to the TLR4/MD2 receptor complex initiating a signaling cascade that leads to the activation of nuclear factor-κB (NF-κB) promoting the transcription of inflammatory cytokines.^88^ MIT in mice resulted in a decreased abundance of *Proetus,* potentially indicating a positive effect on obesity-associated low-grade inflammation.^42^ In humans, two weeks of MIT and HIIT reduced intestinal inflammation, altered the gut microbiome (increased Bacteroidota (*P*), *Veillonella* (G), and decreased *Blautia* spp.), and decreased endotoxemia in insulin-resistant individuals.^87^ In contrast, 10 weeks of M-HIT training did not significantly change systemic inflammatory markers in overweight females, although *Bifidobacterium* abundance was reported to be negatively associated with circulating IL-6 levels.^58^

Long-term MIT-HIIT training increased expression of tight junction proteins, such as claudin, occludin, and ZO-1, that regulate intestinal permeability.^29^ MIT (10-12 weeks) in obese mice increased expression of colonic occludin and ZO-1 and significantly mitigated the HFD-induced reduction in goblet cells,^29^^,^^44^ with HIIT eliciting a more pronounced increase in ZO-1 expression.^29^ These findings suggest that exercise intensity may influence inflammation partly through microbiota-mediated improvements in barrier integrity.

### Gastrointestinal physiology, host metabolites, and sampling considerations

Mechanistically, exercise alters gastrointestinal physiology in intensity-dependent ways.^89^ Exercise modifies transit time and luminal pH, shaping microbial growth conditions. Acute and high-intensity exercise redistributes blood flow away from the splanchnic circulation, including the intestines, increasing permeability and gastrointestinal distress, which may transiently alter luminal oxygen, substrate availability, and microbial ecology.^90^ Accordingly, the timing of stool sample collection relative to the last exercise bout is critical. Samples collected shortly after an exercise session may capture acute, transient shifts in microbial composition, metabolite concentrations, or markers of intestinal permeability driven by hypoperfusion, stress hormones, or altered transit, whereas samples collected ≥24–48 h post-exercise better reflect chronic training adaptations.^61^^,^^90^ Failure to standardize or report sampling timing may confound interpretation and contribute to variability across studies. Most animal studies and all human studies within this review reported sample collection at ≥24 h post-exercise.

Host-derived metabolites further link exercise intensity to microbial remodeling. Exercise-induced lactate production may represent an important host-microbiome signaling axis^91^^,^^92^ since lactate can enter the intestinal lumen from circulation and serve as a substrate for bacteria that convert lactate into SCFAs, such as butyrate.^93^ Petriz et al., reported negative associations between *Clostridiaceae* (F), *Bacteroidaeae* (F) *Ruminococcus* (G), and blood lactate, whereas *Oscillospira* (G) demonstrated a positive correlation in a Zucker rat model undergoing 4-weeks of MIT,^55^ suggesting lactate-driven cross-feeding dynamics. Similarly, higher-intensity exercise, a low-carbohydrate diet, or fasted exercise increases circulating ketone bodies, including *β*-hydroxybutyrate (BHB), a signaling metabolite that improves inflammation and oxidative stress.^94^^,^^95^ Emerging evidence suggests that ketone bodies may also influence gut microbial composition and intestinal barrier function, either directly through luminal exposure or indirectly via host metabolic and immune pathways.^96^^,^^97^ Together, intensity-dependent shifts in gastrointestinal physiology, substrate flux, and host metabolite availability provide plausible mechanistic pathways linking exercise to microbiome remodeling and downstream metabolic adaptations.

### Interindividual variability and translational implications

Despite consistent themes, substantial interindividual variability exists. For example, only about half of women with overweight/obesity demonstrated microbiome responsiveness to training,^60^ underscoring the potential for exercise-responsive taxa and the influence of host-specific factors such as baseline microbiota, diet, immune status, sex, and age.^98^^,^^99^ While Ortega-Santos et al. reported similar responses between male and female animals,^100^ others suggest that sex hormone-microbiota interactions, differences in body composition, and colonic transit time may drive sex-specific effects.^99^ Longitudinal designs with stratification by sex, age, and baseline microbiota are needed to distinguish causal from context-dependent effects.

Finally, microbiota-targeted strategies such as probiotics, postbiotics, or fecal microbiota transplantation (FMT) may complement exercise. FMT from exercise-trained mice improved body weight, adiposity, and inflammation in obese recipients, which can be attributed to shifts in taxa such as *Odoribacter*, *Helicobacter*, and *AF12.*^47^ While promising, validation in human populations is needed, and future research should identify key exercise-responsive microbes and elucidate the contributions of co-transferred components, including microbial metabolites.

Collectively, these findings highlight the critical need for integrative mechanistic, longitudinal, and translational investigations to delineate the pathways through which exercise intensity modulates gut microbial composition and function, and how these adaptations influence host metabolic health, particularly in the context of obesity and related metabolic disorders.

## Data sharing statement

Data sharing not applicable—no new data generated.
